# Anticancer Activities of Polyynes from the Root Bark of *Oplopanax horridus* and Their Acetylated Derivatives

**DOI:** 10.3390/molecules19056142

**Published:** 2014-05-14

**Authors:** Lan-Zhen Meng, Wei-Hua Huang, Chong-Zhi Wang, Chun-Su Yuan, Shao-Ping Li

**Affiliations:** 1 State Key Laboratory of Quality Research in Chinese Medicine, Institute of Chinese Medical Sciences, University of Macau, Avenida da Universidade, Macau, China; E-Mail: Meng-lz@hotmail.com (L.-Z.M.); endeavor34852@aliyun.com (W.-H.H.); 2 Tang Center for Herbal Medicine Research, The Pritzker School of Medicine, University of Chicago, 5841 South Maryland Avenue, MC 4028, Chicago, IL 60637, USA; E-Mail: CWang@dacc.uchicago.edu (C.-Z.W.)

**Keywords:** polyynes, *Oplopanax*, anticancer, structure-activity relationship

## Abstract

Six polyynes **OH-1**~**6**, some of which are occur naturally in acetylated form, had been isolated and identified from the root bark of *Oplopanax horridus* (Devil’s Club), a natural dietary supplement and medicinal plant in North America. During the evaluation of the polyynes’ potential anticancer activities, sixteen more acetylated derivatives **OHR-1**~**16** have synthesized and their anti-proliferation activity on MCF-7, MDA-MB-231, A549, HepG2 and LO2 cells assayed to elucidate their structure-activity relationships. The results showed that **OH-1** ((3*S*, 8*S*)-falcarindiol) had the most potent anticancer activity, with IC_50_ values of 15.3, 23.5, 7.7 and 4.7 μM on MCF-7, A549, HepG2 and MDA-MB-231 cells, respectively. For the primary structure-activity relationship, the anticancer activities of polyynes become weaker if their hydroxyl groups are acetylated, the terminal double bonds transformed into single bonds or they contain one more methylene group in the main skeleton chain.

## 1. Introduction

Polyynes, namely polyacetylenes, have been found in many food and dietary plants [1,2,3,4,5]. To date, pharmacological investigations have revealed that polyynes from natural sources show antibacterial, anti-fungal, antiinflammatory, nerotoxic and anticancer activities [6,7,8,9]. Polyynes were regarded to be responsible for many bioactivities of *Oplopanax horridus*, which has long history of medicinal use and as a dietary supplement. *O. horridus* was well-known and marketed in North America as Devil’s Club extracts, which are also edible as a respiratory stimulant and expectorant, and used for rheumatoid arthritis, autoimmune conditions, eczema, type II diabetes, external and internal infections [10,11,12,13,14,15].

Recent studies have revealed that four purified polyynes from *O. horridus* showed potential anticancer activities [16,17,18]. All these polyynes possess hydroxy groups in their structures, and two of them, which occured naturally in acetylated form had weaker anti-proliferation effects. The primary hypothesis based on the anti-proliferation investigation of only these four polyynes on the selected cancer cell lines was that acetylation of polyynes had a negative contribution to their anticancer activities. However, as the two acetylated polyynes had 18 carbon atoms in the main skeleton chain (C18 polyynes) while the others had 17 carbon atoms as the main structural chain (C17-polyynes). It seems that they could not be compared diretly together to reach that conclusion [17].

**Figure 1:**
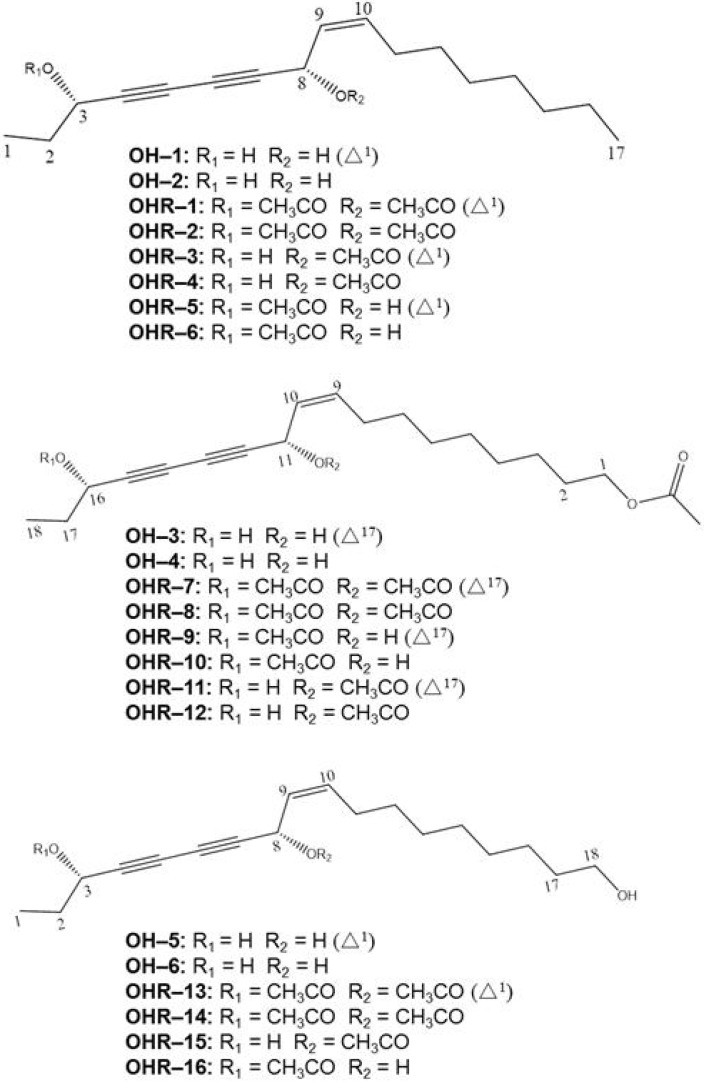
The structures of the isolated and derivative polyynes.

During the course of discovering interesting anticancer molecules from the title plant, six polyynes **OH-1~OH-6** (Figure 1) had been purified from hydrophobic parts of the herbal medicine extracts and identified. Some of these compounds had displayed anti-proliferation activity against certain cancer cell lines [17,19]. Among the polyynes, (3*S*,8*S*)-falcarindiol had been obtained separately from (3*R*, 8*S*)-falcarindiol, which was also reported to have anticancer activities [20]. Since two of the polyynes from *O. horridus* naturally occur acetylated, sixteen more acetylated derivatives **OHR-1~OHR-16** (Figure 1) have now been synthesized for the evalutation of the polyynes’ potential anticancer activities. The possible mechanisms and structure-function relationships in anti-proliferative activity were also studied.

## 2. Results and Discussion

### 2.1. Chemistry

The known compounds were identified by comparing their physical and spectroscopic data with values reported in the literature. The structures of acetylated compounds were characterized by IR, ^1^H-NMR, ^13^C-NMR, and HMBC spectra. Especially, the position of the acetyl group in the compounds was elucidated and fixed by the HMBC spectra. The purity (≥96%) of the target compounds was verified by HPLC.

Six polyynes **OH-1~OH-6** had been isolated and purified from the root bark of *O. horridus*. Acetylated polyynes **OHR-1~OHR-16** were synthesized from **OH-1~OH-6** with acetic anhydride in ethyl acetate with sodium carbonate added to the stirring mixtures. After quenching the reactions, the reaction mixture was cooled to room temperature and evaporated to remove the organic solvents. The residue was diluted with water and then extracted with chloroform. The chloroform layer was evaporated to remove the organic solvents. After that, the acetylated products were subjected to chromatographic separation to obtain the derivatives **OHR-1~OHR-16**.

### 2.2. Effects of the 22 Polyynes on Proliferation of Selected Cancer Cells

The polyynes isolated from *O. horridus* have been evaluated for antiproliferative effects on some selected cancer cell lines such as human breast cancer cell line MCF-7, non-small cell lung cancer (NSCLC) cells, human colorectal cancer cell lines HCT-116 and SW-480 [21,22,23]. As shown in Table 1, the 22 polyynes exhibited different antiproliferative effects on the human breast cancer MCF-7, human lung adenocarcinoma epithelial A549, liver hepatocellular HepG2 and human breast cancer µM-231 cell lines. At the adopted concentrations (1–300 µM), polyynes **OHR-7~8** and **OHR-13~16** were not observed to inhibit the cancer cell growth of any of the four cell lines. **OHR-13** and **OHR-14** showed some antiproliferative effects on MDA-MB-231 cancer cells at about 150 µM, but such effects were not observed in other cancer cell lines. Moreover, other polyynes showed potentially different cell growth inhibition of the four cancer cell lines.

**Table 1:** Anticancer activities of polyynes from *O. horridus* and their acetylated derivatives.

Polyynes	IC_50_ (μM)				
	A549	MCF-7	HepG2	LO2	MDA-MB-231
**OH-1**	23.5 ± 4.1	15.3 ± 0.3	7.7 ± 1.3	8.7 ± 0.5	4.7 ± 1.4
**OH-2**	44.1 ± 9.4	27.5 ± 2.2	38.4 ± 7.5	30.2 ± 4.8	14.7 ± 2.7
**OH-3**	98.0 ± 6.3	93.6 ± 6.1	47.2 ± 5.9	90.3 ± 6.4	16.5 ± 2.2
**OH-4**	109.9 ± 0.8	100.0 ± 7.0	69.1 ± 1.8	91.9 ± 13.4	87.8 ± 7.9
**OH-5**	150-300	94.4 ± 7.3	48.3 ± 5.9	91.0 ± 9.5	49.0 ± 10.9
**OH-6**	85.7 ± 13.2	106 ± 13.2	45.3 ± 6.3	90.8 ± 4.0	55.7 ± 11.8
**OHR-1**	>300	>300	114.9 ± 17.1	>300	13.9 ± 3.4
**OHR-2**	24.7 ± 5.6	36.9 ± 4.8	6.6 ± 1.5	32.1 ± 9.0	4.8 ± 0.5
**OHR-3**	31.4 ± 4.3	25.8 ± 5.8	31.5 ± 2.1	31.5 ± 4.0	6.6 ± 0.8
**OHR-4**	33.3 ± 8.5	28.0 ± 2.9	59.5 ± 3.1	34.3 ± 1.5	23.4 ± 3.2
**OHR-5**	30.1 ± 4.4	30.6 ± 3.1	43.7 ± 6.6	31.1 ± 1.8	13.9 ± 5.8
**OHR-6**	97.5 ± 8.2	59.6 ± 7.5	38.5 ± 6.2	46.1 ± 11.7	36.2 ± 13.4
**OHR-7**	>300	>300	>300	>300	>300
**OHR-8**	>300	>300	100–300	>300	19.6 ± 6.9
**OHR-9**	83.1 ± 8.4	81.3 ± 1.0	59.9 ± 3.0	86.2 ± 11.3	52.0 ± 7.5
**OHR-10**	100.9 ± 7.3	94.3 ± 6.1	78.4 ± 6.5	100–300	56.7 ± 6.9
**OHR-11**	98.2 ± 17.5	78.1 ± 3.4	77.0 ± 9.5	93.1 ± 14.0	51.5 ± 8.4
**OHR-12**	100–300	90.7 ± 0.3	100–300	100–300	77.7 ± 1.9
**OHR-13**	>300	>300	>300	>300	137.0 ± 28.4
**OHR-14**	>300	>300	150–300	>300	155.5 ± 35.5
**OHR-15**	>300	>300	>300	>300	100–300
**OHR-16**	>300	>300	>300	>300	>300

In A549 cells, **OH-3~6** and **OHR-2**, **5**, **9~11** showed moderate antiproliferative effects, with IC_50_s of more than 80 µM. Cell growth of A549 could be inhibited by 50% by **OH-1~2** and **OHR-3~4**, **6** at the concentrations of 23.5 ± 4.1 µM, 44.1 ± 9.4 µM, 30.1 ± 4.4 µM 31.4 ± 4.3 µM and 33.3 ± 8.5 µM, respectively (Table 1). **OH-1** and **OHR-3** showed stronger effects in that A549 cell growth was inhibited by 95.6% and 90.8%, respectively, when administrated at 60 µM (both *p* < 0.01). In A549 cells, **OH-1** and **OHR-3** showed the most potent antiproliferative effects.

Similar results were also observed in MCF-7 cells. **OH-3~6** and **OHR-5**, **9~11** also exhibited moderate antiproliferative effects as in A549 cells. The IC_50_ values of **OH-1~2** and **OHR-3~4**, **6** were observed at the concentration of 15.3 ± 0.3 µM, 27.5 ± 2.2 µM, 30.6 ± 3.1 µM, 25.8 ± 5.8 µM and 28.0 ± 2.9 µM, respectively, which were lower on this cell line than those of A549 cells (Table 1). Antiproliferative effects of other polyynes on MCF-7 cells were almost the same with that on A549 cells. Moreover, **OH-1** showed the most potent antiproliferative effects in this cell line.

In HepG2 and MM–231 cells, **OH-4** and **OHR-9~11** displayed moderate antiproliferative effects on the two cancer cell lines at lower concentrations than those of A549 and MCF-7 cell lines (Table 1). The IC_50_ values of **OH-1~3**, **5~6** and **OHR-3~6** were observed much lower on this cell lines than those of A549 and MCF-7 cells (Table 1). Among all the polyynes, **OH-1** also showed the most potent antiproliferative effects in these two cell lines, with IC_50_ values of 7.7 ± 1.3 µM and 4.7 ± 1.4 µM, respectively.

### 2.3. Anti-Proliferative Activity and Possible Structure-Activity Relationships

Anti-proliferative tests of the six isolated polyynes **OH-1~OH-6** and their derivatives **OHR-1~OHR-16** were conducted on four cancer cell lines and a normal human hepatic cell line. As shown in Table 1, the 22 compounds exhibited different extents of anti-proliferative activity on the cancer cell lines with various IC_50_ values.

**Figure 2:**
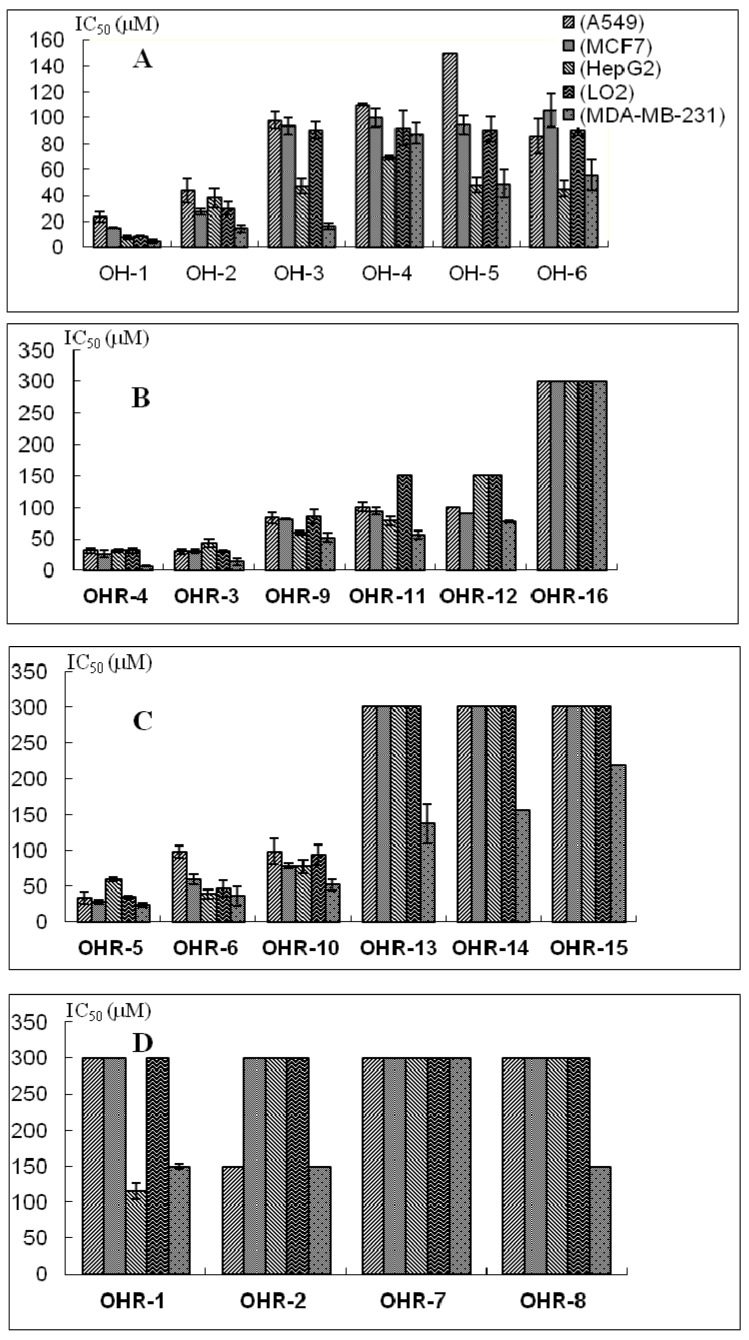
The anti-proliferative effects of the similar polyynes on MCF-7 cells, A549 cells, HepG2 cells and MDA-MB-231 cells. (**A**) all the natural polyynes; (**B**) the polyynes with the hydroxyl in the middle of the carbon chain displaced; (**C**) the polyynes with the hydroxyl near the end of the carbon chain; (**D**) the polyynes with no hydroxyls.

The biological activities of the polyynes with same structure features were compared (Figure 2). In Figure 2A, the anti-proliferation effects of all the isolated compounds **OH-1~OH-6** showed that **OH-1**, **OH-3** and **OH-5** had stronger activities than **OH-2**, **OH-4** and **OH-6** on all the investigated cancer cell lines, except for OH-5 and OH-6 on A549 cells, respectively. The **OH-5** and **OH-6** effects on A549 cells deviated, possibly due to different mechanisms on anticancer activity of this cell line. Additionally, **OH-1** and **OH-2** containing 17 carbon atoms in the main chain (C17-polyynes) displayed higher activity than other polyynes consisting of 18 carbon atoms as the structure skeleton chain (C18- polyynes).

In Figure 2B, the acetylated polyynes **OHR-3**, **OHR-9** and **OHR-12** show higher activities than **OHR-5**, **OHR-10** and **OHR-16** on all the cancer cell lines. These polyynes had their acetyl groups connected with the hydroxyls in the middle of the carbon chain. Among them, **OHR-3** and **OHR-5** derivatizated from **OH-1** and **OH-2**, respectively, possessed higher inhibition than other acetylated C18-polyynes. In Figure 2C, the acetylated polyynes **OHR-4** and **OHR-11** possessed better activities than **OHR-6** and **OHR-11** toward all the cancer cell lines except A549 cells, respectively. **OHR-13**, **OHR-14** and **OHR-15** even exhibited no cytotoxicity on any of the selected cancer cell lines except weak activity on MDA-MB-231 cells (IC_50_ > 120 μM). These polyynes synthesized with the acetyl group connected with the hydroxyl near the end of the carbon chain were compared with the ones in Figure 2B to possibly conclude that the hydroxyl at the end of the carbon chain contributed more to the anticancer activity than the hydroxyl in the middle of the carbon chain. In Figure 2D, **OHR-1**, **OHR-2**, **OHR-7** and **OHR-8** almost showed no cytotoxicity toward most of the cancer cell lines when all the hydroxyls in these polyynes were substituted, although **OHR-1** and **OHR-8** had weak effects on MCF-7 and HepG2 cells with IC_50_ > 150 µM.

For these similar polyynes, the anti-proliferation effects data showed that the compounds with terminal ethylenic bonds exhibited higher activity compared to the polyynes with terminal single bond, without consideration of the ones (IC_50_ ≥ 300 μM); The C-17 polyynes possesed stronger activities than the C18-polyynes, which has one more methylene group than the C17-polyynes except the polyynes with IC_50_ values more than 300 μM. In addition, hydroxypolyynes had higher activity than acylated polyynes. The primary structure–activity analysis thus showed that the observed inhibitions were influenced mostly by the carbon chain length, terminal ethenyl, hydroxyl groups and acylations of polyynes.

Moreover, the compounds’ effects on the human normal hepatic cells was investigated to evaluate their potential hepatotoxicity *in vitro*. It was found that the compounds with higher anti-proliferative activity presented more potential cytotoxic effect on normal human hepatic cells as well (Table 1), which means these compounds may have potential hepatotoxicity *in vivo*. Actually, the safety and efficiency of *O. horridus* have not been evaluated totally thus far though it has a long history of use medicinally and as a dietary supplement [21,24], so more studies are definitely needed. 

### 2.4. Apoptosis and Cell Cycle Assays

**Figure 3:**
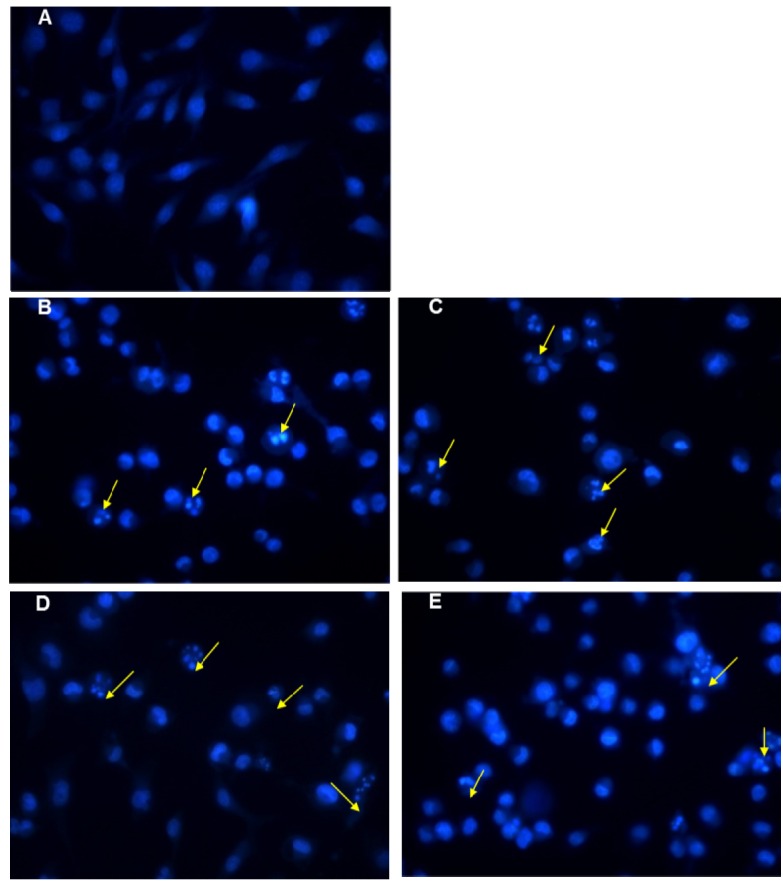
Detection of apoptosis in MDA-MB-231 cells by Hochest-staining. Treated cells were stained with Hoechst 33342, and then observed under a fluorescent microscope (magnification of ×400). (**A**) Sovent control treated MDA-MB-231 cells; (**B–E**) **OHR-1** (15 μM), **OHR-2** (5 μM), **OHR-3** (10 μM) and **OHR-5** (15 μM) treated MDA-MB-231 cells for 12h. The yellow arrows indicated classic apoptosis characteristics in cells with morphology change, chromatin condensation and apoptotic bodies.

Next, cell cycle analysis and apoptosis assays were performed on the cancer cells. Previous work had reported that **OH-1~4** exhibited potential anticancer acitives on human breast cancer and colon cancers through cell arrest in G2/M phase and induction of appoptosis at both earlier and later stages [17]. In this study, the potential mechanisms of the four new compounds **OHR-1**, **-2**, **-3** and **-5** with the strongest anti-proliferative activity among the series of acylated polyynes were investigated. It was found that these four acylated polyynes could induce obvious apoptosis in MM–231 cells, which showed classical apoptotic morphology, chromatin condensation and apoptotic bodies (Figure 3) with Hoechst staining.

**Figure 4:**
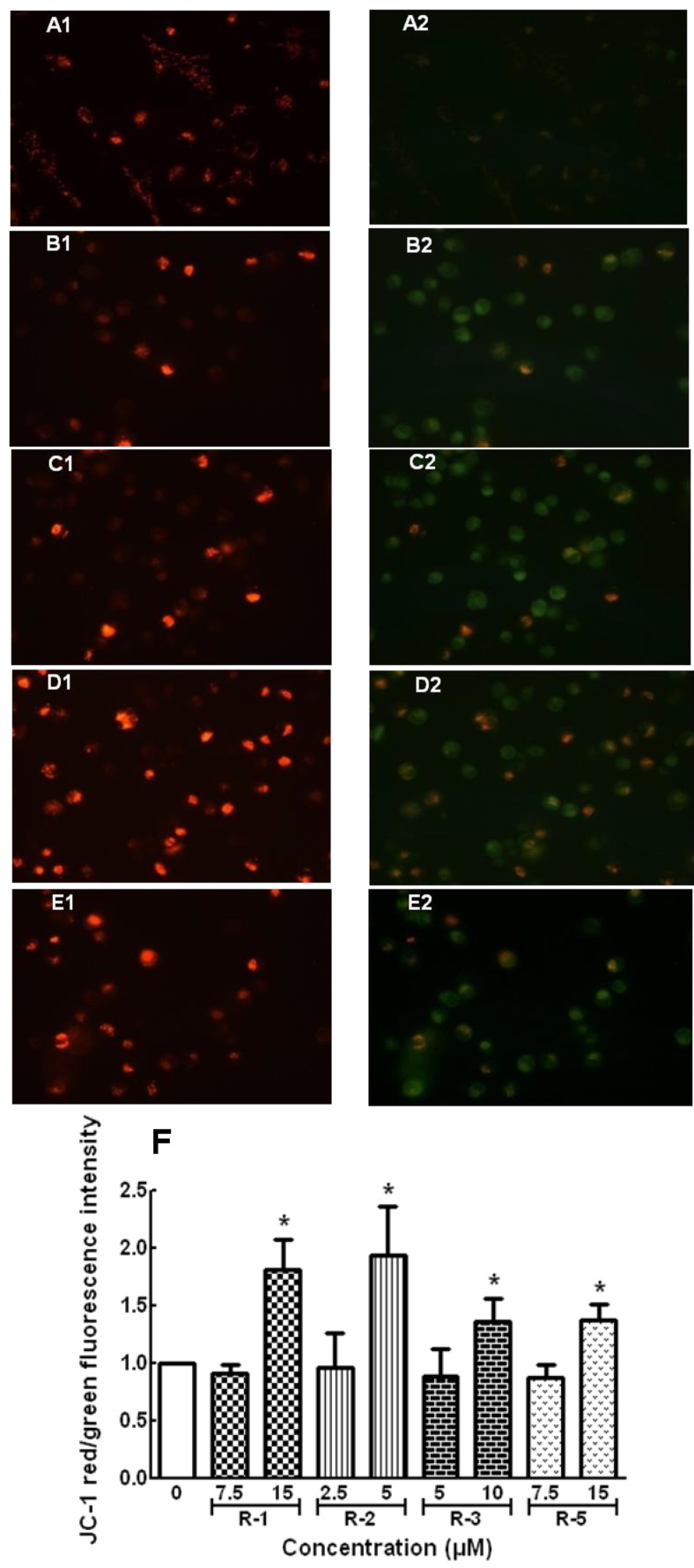
Effect of **OHR-1**, **-2**, **-3** and **-5** on mitochondrial potential in MDA-MD-231 cells. Treated cells stained by JC–1 dye and observed by fluorescence microscopy (magnification of ×400). (**A1**, **A2**) Solvent control-treated cells showed most of cells had stronger J-aggregation stained red fluorescence. (**B1-E1**, **B2-E2**) **OHR-1** (15 μM), **OHR-2** (5 μM), **OHR-3** (10 μM) and **OHR-5** (15 μM) treated MDA-MB-231 cells show a majority of cells stained green fluorescence due to low △Ψm. (**F**) Analysis of the △Ψm (ration of green/red fluorescence intensity) in cells measured by flow cytometry. The data were expressed as ratio of Δψm between **OHR-1**, **-2**, **-3** and **-5** treatments and control cells (solvent vehicle set at 100%). * *p* < 0.05 *vs*. vehicle control.

**Figure 5:**
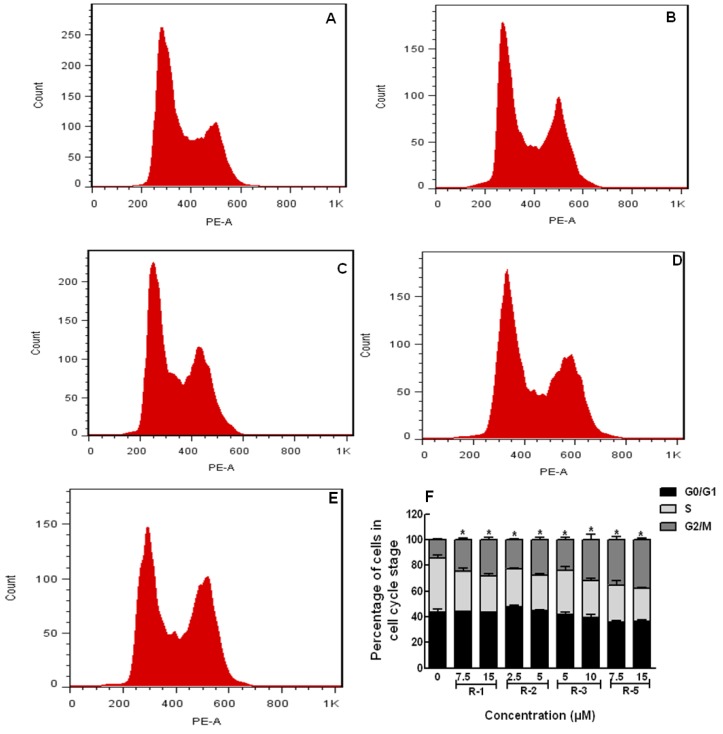
Cell cycle analysis of MDA-MB-231 cells after exposure to **OHR-1**, **-2**, **-3** and -**5** for 24 h. After treatment, cells were fixed in 70% ethanol and then analyzed by flow cytometry distribution by propidium iodide/DNA content staining. (**A**) Solvent control- treated MDA-MB-231 cells; (**B1-E1**) Cell cycle analysis of cells treated with **OHR-1** (7.5 μM), **OHR-2** (2.5 μM), **OHR-3** (5 μM) and **OHR-5** (7.5 μM) for; (**B2-E2**): Cell cycle analysis of cells treated with **OHR-1** (15 μM), **OHR-2** (5 μM), **OHR-3** (10 μM), **OHR-5** (15 μM) treated MDA-MB-231 cells. (**F**) Cell cycle analysis of MDA-MB-231 cells treated with **OHR-1**, **-2**, **-3** and **-5** measured by flow cytometry. * *p* < 0.05 *vs*. vehicle control.

On the other hand, JC–1 dye was applied to test the Δψm, which is an important parameter of mitochondrial function used as an indicator of cell health. In apoptotic cells with low Δψm, JC–1 remained in the monomeric form, and showed only green fluorescence in cells. The results showed that **OHR-1**, **-2**, **-3** and **-5** at high concentration could induce MM–231 cells apoptosis by decreasing the Δψm, indicated by a change color from red to green fluorescence (Figure 4A–E) and increasing green to red fluorescence intensity ratio (Figure 4F). Moreover, their influences on the MM–231 cell cycle were determined by PI staining and flow cytometry analysis. It was observed that **OHR-1**, **-2**, **-3** and **-5** could arrest MM–231 cells in G2/M phases by 14.4% ± 3.9% compared to the solvent control. The arrest rates at low conentration were 24.6% ± 3.6%, 22.6% ± 2.6%, 24.2% ± 2.9% and 33.2% ± 4.6%, repectively, while at high concentration they were 31.7% ± 3.2%, 30.4% ± 3.1%, 25.6% ± 7.5% and 38.0% ± 1.7%, repectively, as shown in Figure 5.

These four polyynes did not exhibit significant effects in the G-phase. Therefore, we regard that induction of apoptosis and cell cycle arrest by **OHR-1**, **-2**, **-3** and **-5** contributed to their anti-proliferative effects on MM–231 cells. Acetylated polyynes have the same mechanism as non-acetylated polyynes in the anti-proliferative activity on the cancer cells.

For this investigation, six polyynes and 16 acetylated polyynes were evaluated for their anticancer activities toward selected human cancer lines. **OH-1**, which has 17 carbon atoms in the main chain with no acetyl group and a terminal ethenyl group, showed the highest anticancer activities in all tested cell lines. This study also suggested that polyynes from *O. horridus* may possibly be active anticancer ingredients.

## 3. Experimental Section

### 3.1. General Procedures

Optical rotations were recorded on a Perkin Elmer Model 341 polarimeter. UV spectrum was measured on a Beckman Coulter DU 640 spectrophotometer. IR spectra were obtained with a PerkinElmer Spectrum 100 FT-IR spectrometer with KBr pallets. The ^1^H-, ^13^C-, and 2D-NMR spectra were tested on a Bruker AV-500 spectrometer (*δ* in ppm, *J* in Hz) with tetramethylsilane (TMS) as an internal standard (Bruker, Germany). ESI-MS and HR-ESI-MS measurements were carried out on an Agilent 1100 series LC/MSD Trap VL mass spectrometer and an Agilent time-of-flight (TOF) mass spectrometer respectively (Agilent, Santa Clara, CA, USA). Silica gel (100–200 and 200–300 mesh) (Qingdao Haiyang Chemical Co. Ltd, Qingdao, China) and Merck Reversed-phase C_18_ (RP-C_18_) silica gel (40–75 µm) (Merck, Darmstadt, Germany) were used for column chromatography (CC). Precoated silica gel GF_254_ plates (Qingdao Haiyang Chemical Co. Ltd, Qingdao, China) were used for TLC. Supercritical fluid extraction was manipulated on a supercritical fluid extractor (SFT-250, Thar Instruments, Inc., Pittsburgh, PA, USA). Analytical HPLC was performed on an Agilent 1200 liquid chromatograph with an Agilent Zorbax SB RP-C_18_ column (250 mm × 4.6 mm inside diameter (I.D.), 5 µm, Agilent). Preparative HPLC was carried out with an Agilent 1100 liquid chromatograph with a Phenomenex Luna RP-C_18_ column (250 mm × 21.2 mm I.D., 5 µm).

### 3.2. Plant Material

The dried roots bark of *O.* *horridus* was obtained from Pacific Botanicals Co. Ltd (Chicago, IL, USA) and authenticated by one of the authors (C.-Z. Wang) in March, 2012. A voucher specimen (Lot: OHR-20120926-1) has been deposited in the Institute of Clinical Pharmacology, Central South University, Hunan Province, China.

### 3.3. Chemicals

HPLC-grade methanol and acetonitrile were purchased from Merck. The deionized water used for HPLC was purified by a Milli-Q purification system (Millipore, Billerica, MA, USA). Chloroform, Methanol and Ethyl acetate (analytical grade) was obtained from Beijing Chemical Reagent Plant (Beijing, China). All the liquid and solid reagents were purchased from Sigma (St. Louis, MO, USA).

### 3.4. Extraction and Isolation

The roots bark of *O. horridus* (7.2 kg) was sieved by a 20 mesh crib after pulverization. Methanol (360 L) was selected to extract the pulverized powder with infusion for 7 days × 3. Then, the methanol extract (900 g) was diffused into pure water (2 L) and extracted with ethyl acetate (EtOAc) and *n*-butanol to yield the corresponding fractions E (312 g) and B (320 g), respectively. The two organic solvents were used for extraction with the volume of 3 L × 3, resp., after which were saturated using pure water. The EtOAc-soluble fraction E (260 g) was separated by silica gel (100–200 mesh) column chromatography (CC) eluted with a gradient of CHCl_3_–MeOH (50:1 to 0:1) to give ten fractions (E1–E10). Fraction E7 (53 g) was then subjected to silica gel (200–300 mesh) CC eluting with CHCl_3_–MeOH (10:1, 8:1 and 5:1) to give six subfractions (E7a–E7f). Subfraction E7d (38 g) was chromatographed on RP-C_18_ silica gel (MeOH–H_2_O, 70:30), then subjected to Prep-HPLC (MeOH–H_2_O, 78:22) to afford **OH-1** (1.2 g) and **OH-2** (2.4 g). Fraction E8 (75 g) was subjected to silica gel (200–300 mesh) CC eluted with CHCl_3_–MeOH (10:1, 6:1 and 4:1) to afford five subfractions (E8a–E8e). Subfraction E8d (45 g) was chromatographed on RP-C_18_ silica gel (MeOH–H_2_O, 67:33), then repeatedly purified by prep-HPLC (MeOH–H_2_O, 70:30) to afford **OH-3** (2.9 g) and **OH-4** (3.2 g). Subfraction E9 (68 g) was further separated by CC on silica gel (200–300 mesh) eluted with CHCl_3_–MeOH (8:1, 5:1 and 3:1) to yield six subfractions (E9a–E9f). Subfraction E9e (36 g) was further purified by prep-HPLC (MeOH–H_2_O, 65:35) to afford **OH-5** (1.8 g) and **OH-6** (2.1 g).

### 3.5. Synthesis of Acetylated Polyynes

A common acetylation method was used. Acetylated polyynes **OHR-1~OHR-16** were synthesized by mixing the subfractions E7d (7.5 g), E8d (7.8 g) and E9e (7.8 g) with acetic anhydride (60 mmol) and Na_2_CO_3_ (6.36 g) in ethyl acetate (20 mL), respectively. The reaction mixture was refluxed for 2 h at 60 °C while the progress of each reaction was monitored by TLC and HPLC. After quenching the reaction, the mixture was cooled to room temperature and evaporated to remove organic solvents under diminished pressure at 50 °C. The residue was diluted with H_2_O (50 mL) and each one was extracted with CHCl_3_ (40 mL × 3). The CHCl_3_ layer was evaporated and the acetylated products were subjected to chromatographic separation to obtain polyyne derivatives **OHR-1~OHR-16** in different yields. Repeated pre-HPLC separation afforded the pure products.

After the subfraction E7d (7.5 g) reacted with acetic anhydride, all the reactants and reaction products were subjected to RP-C_18_ silica gel CC (MeOH–H_2_O, 70:30~100:0) to obtain four subfractions (E7dR1–E7dR4). Subfraction E7dR4 (2.0 g) was purified by prep-HPLC (acetonitrile–H_2_O, 80:20) to give **OHR-1** (200 mg) and **OHR-2** (150 mg). Subfraction E7dR3 (880 mg) was purified by prep-HPLC (acetonitrile–H_2_O, 77:23) to yield **OHR-3** (52 mg) and **OHR-4** (50 mg). Subfraction E7dR2 was further purified by prep-HPLC (acetonitrile–H_2_O, 75:25) to obtain **OHR-5** (28 mg) and **OHR-6** (42 mg).

After the reaction between E8d (7.8 g) and acetic anhydride was finished, the condensate was separated by RP-C_18_ silica gel CC (MeOH–H_2_O, 65:35~100:0) to afford five subfractions (E8dR1~E8dR5). Subfraction E8dR5 (1.8 g) was purified by prep-HPLC (acetonitrile–H_2_O, 78:22) to yield **OHR-7** (180 mg) and **OHR-8** (170 mg). Subfraction E8dR4 (752 mg) was subjected to prep-HPLC (acetonitrile–H_2_O, 74:26) to afford **OHR-9** (112 mg) and **OHR-10** (126 mg). Subfraction E8dR3 (610 mg) was separated by prep-HPLC (acetonitrile–H_2_O, 70:30) to afford **OHR-11** (36 mg) and **OHR-12** (82 mg).

The subfraction E9e (9.2 g) was quenched after reaction with acetic anhydride, then the condensate was chromatographed on RP-C_18_ silica gel (MeOH–H_2_O, 55:45~100:0) to give six subfractions (E9eR1~E9eR5). Subfraction E9eR3 (720 mg) was purified by prep-HPLC (acetonitrile–H_2_O, 58:42) to afford **OHR-13** (24 mg) and **OHR-14** (35 mg). Subfraction E9eR2 (400 mg) was separated by prep-HPLC (acetonitrile–H_2_O, 50:50) to afford **OHR-15** (12 mg) and **OHR-16** (24 mg).

### 3.6. Chemical Characteristics of Compounds ***OH–1~OH-6*** and ***OHR–1~OHR-16***

*(3S,8S,Z)-Heptadeca-1,9-dien-4,6-diyne-3,8-diol* (*(3S,8S)-falcarindiol)* **OH-1**): yellowish oil (1.6 g); 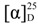 + 213.5° (*c* = 0.36, CHCl_3_); UV (CHCl_3_) λmax (log ξ): 217 (0.81), 243 (1.39), 260 (1.75) and 286 (2.11) nm; IR (KBr) ν_max_: 3345, 3020, 2932, 2850, 2251, 1466, 1021, 938 and 875 cm^−1^; ^1^H-NMR (500 MHz, CDCl_3_): *δ*_H_ 0.88 (3H, t, 7.0, H-17), 1.28 (8H, m, H-13, H-14, H-15, H-16), 1.38 (2H, m, H-12), 2.10 (2H, q, 7.0, H-11), 4.94 (1H, brd, 5.0 Hz, H-3), 5.20 (1H, d, 8.5 Hz, H-8), 5.25 (1H, d, 10.0 Hz, H-1a), 5.46 (1H, ddt, 11.5, 7.5, 1.0 Hz, H-10), 5.50 (1H, ddt, 11.5, 5.0, 1.5 Hz, H-9), 5.62 (1H, dd, 17.5, 1.5 Hz, H-1b), 5.92 (1H, ddd, 17.5, 10.0, 6.0 Hz, H-2). ^13^C-NMR (125 MHz, CDCl_3_): *δ*c 14.0 (C-17), 22.6 (C-15), 27.6 (C-11), 29.0 (C-12), 29.1 (C-14), 29.2(C-13), 31.7(C-16), 58.4 (C-8), 63.3 (C-3), 68.6 (C-6), 70.2 (C-5), 78.3 (C-4), 79.8 (C-7), 117.2 (C-1), 127.6 (C10), 134.4 (C-9), 135.8 (C-2).

*(3S,8S,Z)-Heptadeca-9-en-4,6-diyne-3,8-diol* (*oplopandiol,* **OH-2**): yellowish oil (2.5 g); 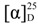 + 208.6° (*c* = 0.28, CHCl_3_); UV (CHCl_3_) λmax (log ξ): 210 (1.25), 245 (1.39), 253 (2.35) and 267 (4.12) nm; IR (KBr) ν_max_: 3350, 3015, 2934, 2860, 2250, 1468, 1025, 940 and 866 cm^−1^; ^1^H-NMR (500 MHz, CDCl_3_): *δ*_H _0.88 (3H, t, 7.0 Hz, H-17), 1.00 (3H, t, 7.5 Hz, H-1), 1.28 (8H, m, H-13, H-14, H-15, H-16), 1.38 (2H, m, H-12), 1.73 (2H, m, H-2), 2.09 (2H, q, 7.0 Hz, H-11), 4.38 (1H, t, 6.5 Hz, H-3), 5.19 (1H, d, 8.5 Hz, H-8), 5.51 (1H, t, 8.5 Hz, H-10), 5.59 (1H, ddt, 11.0, 7.5, 1.0 Hz, H-9). ^13^C-NMR (125 MHz, CDCl_3_): *δ*c 9.3 (C-1), 14.0 (C-17), 22.5 (C-15), 27.7 (C-11), 29.1 (C-12), 29.1 (C-14), 29.3 (C-13), 30.5 (C-2), 31.7 (C-16), 58.40 (C-8), 63.9 (C-3), 68.7 (C-6), 68.8 (C-5), 79.1 (C-4), 80.7 (C-7), 127.7 (C-10), 134.3 (C-9).

*(11S,16S,Z)-11,16-Dihydroxyoctadeca-9,17-dien-12,14-diynyl acetate* (**OH-3**): yellowish oil (2.6 g); 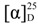 + 180.8° (*c* = 0.53, CHCl_3_); UV (CHCl_3_) λmax (log ξ): 206 (1.76), 235 (1.79), 248 (2.89) and 268 (4.42) nm; IR (KBr) ν_max_: 3410, 2920, 2856, 2246, 1718, 1628, 1560, 1465 and 1025 cm^−1^; ^1^H-NMR (500 MHz, CDCl_3_): *δ*_H_ 1.30 (8H, m, H-3, H-4, H-5, H-6), 1.38 (2H, m, H-7), 1.62 (2H, m, H-2), 2.05 (3H, s, COCH3), 2.10 (2H, q, 7.5 Hz, H-8), 4.06 (2H t, 7.5 Hz, H-1), 4.94 (1H, brd, 5.0 Hz, H-16), 5.19 (1H, d, 8.0 Hz, H-11), 5.24 (1H, d, 10.5 Hz, H-18a), 5.46 (1H, dd, 17.0, 1.5 Hz, H-18b), 5.51 (1H, dd, 10.5, 8.0 Hz, H-9), 5.59 (1H, ddt, 10.5, 7.5, 1.0Hz, H-10), 5.93 (1H, ddd, 17.0, 10.5, 1.5 Hz, H-17). ^13^C-NMR (125 MHz, CDCl_3_): *δ*c 20.9 (COCH_3_), 25.7 (C-2), 27.5 (C-3), 28.5 (C-5), 28.9 (C-4), 29.0 (C-6), 29.1 (C-8), 29.1 (C-7), 58.4 (C-11), 63.2 (C-16), 64.7 (C-1), 68.6 (C-13), 70.0 (C-14), 78.4 (C-15), 80.7 (C-12), 117.0 (C-18), 127.9 (C-10), 134.1 (C-9), 135.9 (C-17), 171.6 (C=O).

*(11S,16S,Z)-11,16-Dihydroxyoctadeca-9-en-12,14-diynyl acetate* (**OH-4**): yellowish oil (3.0 g); 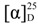 + 168.8° (*c* = 0.47, CHCl_3_); UV (CHCl_3_) λmax (log ξ): 205 (1.88), 233 (1.89), 246 (3.39) and 266 (4.05) nm; IR (KBr) ν_max_: 3440, 2930, 2855, 2250, 1720, 1622, 1550, 1286 and 1020 cm^−1^; ^1^H-NMR (500 MHz, CDCl_3_): *δ*_H_ 1.00 (3 H, t, 7.5 Hz, H-18), 1.30 (8H, m, H-3, H-4, H-5, H-6), 1.38 (2H, m, H-7), 1.62 (2H, m, H-2), 1.73 (2H, m, H-17), 2.05 (3H, s, COCH_3_), 2.11 (2H, q, 7.5 Hz, H-8), 4.05 (2H, t, 7.0 Hz, H-1), 4.37 (1H, t, 6.5 Hz, H-16), 5.18 (1H, d, 8.5 Hz, H-11), 5.50 (1H, ddt, 10.6, 7.5, 1.5 Hz, H-9), 5.58 (1H, ddt, 10.6, 8.5, 1.5 Hz, H-10). ^13^C-NMR (125 MHz, CDCl_3_): *δ*c 9.3 (C-18), 21.0 (COCH_3_), 25.7 (C-2), 27.5 (C-8), 28.9 (C-6), 28.9 (C-5), 29.0 (C-4), 29.1 (C-3), 29.1 (C-7), 30.5 (C-17), 58.4 (C-11), 64.7 (C-16), 68.6 (C-1), 68.7 (C-13), 70.0 (C-14), 79.1 (C-15), 80.7 (C-12), 127.9 (C-10), 134.0 (C-9), 171.6 (C=O).

*(11S,16S,Z)-Octadeca-9,17-dien-12,14-diyne-1,11,16-triol* (*oplopantriol A,* **OH-5**): yellowish oil (1.8 g); 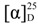 +194.4° (*c* = 0.16, CHCl_3_); UV (CHCl_3_) λmax (log ξ): 226 (1.10), 255 (4.09) and 261 (3.95) nm; IR (KBr) ν_max_: 3357, 3022, 2929, 2855, 2251, 2150, 1675, 1405 and1303 cm^−1^. ^1^H-NMR (500 MHz, CDCl_3_): *δ*_H_ 1.31 (8H, m, H-3, H-4, H-5, H-6), 1.39 (2H, m, H-7), 1.56 (2H, m, H-2), 2.11 (2H, dq, 1.5, 7.5 Hz, H-8), 3.64 (2H, t, 7.0 Hz, H-1), 4.93 (1H, brd, 5.5 Hz, H-16), 5.19 (1H, d, 8.0 Hz, H-11), 5.22 (1H, dt, 10.0, 1.0 Hz, H-18a), 5.46 (1H, dt, 17.4, 1.0 Hz, H-18b), 5.51 (1H, ddt, 10.6, 8.2, 1.5 Hz, H-9), 5.58 (1H, ddt, 10.6, 7.3, 1.5 Hz, H-10), 5.93 (1H, ddd, 17.4, 10.0, 5.5 Hz, H-17). ^13^C-NMR (125 MHz, CDCl_3_): *δ*c 25.6 (C-3), 27.5 (C-8), 28.8 (C-7), 29.0 (C-6), 29.1 (C-4), 29.2 (C-5), 32.6 (C-2), 58.5 (C-11), 63.0 (C-1), 63.3 (C-16), 68.7 (C-13), 70.1 (C-14), 78.5 (C-15), 79.8 (C-12), 117.1 (C-18), 127.9 (C-9), 134.2 (C-10), 136.0 (C-17).

*(11S,16S,Z)-Octadeca-9-en-12,14-diyne-1,11,16-triol* (*oplopantriol B,* **OH-6**): yellowish oil (2.5 g); 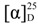 + 233.0° (*c* = 0.3, CHCl_3_); UV(CHCl_3_) λmax (log ξ): 207 (1.07), 232 (1.17), 263 (3.98), and 288 (3.84) nm; IR (KBr) ν_max_: 3355, 3021, 2930, 2856, 2232, 2143, 1656, 1463, 1305, 1095 and 1017 cm^−1^; ^1^H-NMR (500 MHz, CDCl_3_): *δ*_H_ 1.00 (3H, s, H-18), 1.74 (2H, m, H-17),1.31 (8H, m, H-3, H-4, H-5, H-6), 1.38 (2H, m, H-7), 1.57 (2H, m, H-2), 2.11 (2H, dq, 1.5, 7.1 Hz, H-8), 3.64 (2H, t, 6.5 Hz, H-1), 4.37 (1H, t, 6.6 Hz, H-16), 5.19 (1H, d, 8.0 Hz, H-11), 5.52 (1H, ddt, 10.6, 7.5, 1.5 Hz, H-9), 5.58 (1H, ddt, 10.6, 7.3, 1.5 Hz, H-10). ^13^C-NMR (125 MHz, CDCl_3_): *δ*c 9.3 (C-18), 25.6 (C-2), 27.5 (C-8), 28.8 (C-7), 29.0 (C-6), 29.1 (C-4), 29.2 (C-5), 30.6 (C-17), 32.6 (C-2), 58.5 (C-11), 63.0 (C-1), 63.8 (C-16), 68.8 (C-13), 68.8 (C-14), 80.9 (C-15), 79.1 (C-12), 128.0 (C-19), 134.1 (C-10).

*(3S,8S,Z)-Heptadeca-9-en-4,6-diyne-3,8-diyl diacetate* (**OHR-1**): yellowish oil (210 mg); 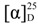 + 202.8° (*c* = 0.86, CHCl_3_); UV(CHCl_3_) λmax (log ξ): 206 (1.27), 232 (1.37), 253 (3.73), and 267 (4.12) nm; IR (KBr) ν_max_: 3012, 2920, 2816, 2248, 1726, 1718, 1612, 1436, 1252, 1068 and 930 cm^−1^; ^1^H-NMR (500 MHz, CDCl_3_): *δ*_H_ 0.88 (3H, t, 7.0, H-17), 1.00 (3H, t, 7.0, H-1),1.28 (8H, m, H-13, H-14, H-15, H-16), 1.38 (2H, m, H-12), 1.79 (2H, m, H-2), 2.07 (6H, s, COCH_3_) 2.13 (2H, dq, 7.0, 1.0 Hz, H-11), 5.33 (1H, t, 6.5 Hz, H-3), 5.46 (1H, ddt, 11.0, 8.5, 1.5 Hz, H-9), 5.66 (1H, dt, 11.0, 7.5, 1.0 Hz, H-10), 6.11 (1H, dt, 8.5, 1.0 Hz, H-8). ^13^C-NMR (125 MHz, CDCl_3_): *δ*c 9.2 (C-1), 14.0 (C-17), 20.8 (COCH_3_), 20.9 (COCH_3_), 22.6 (C-15), 27.8 (C-11), 27.8 (C-12), 29.1 (C-14), 29.1 (C-13), 29.2 (C-2), 31.7 (C-16), 60.1 (C-8), 65.2 (C-3), 69.2 (C-6), 69.3 (C-5), 76.0 (C-4), 77.3 (C-7), 123.8 (C-10), 136.4 (C-9), 169.4 (C=O), 169.7 (C=O).

*(3S,8S,Z)-Heptadeca-1,9-dien-4,6-diyne-3,8-diyl diacetate* (**OHR-2**): yellowish oil (220 mg); 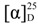 + 243.5° (*c* = 0.92, CHCl_3_); UV (CHCl_3_) λmax (log ξ): 208 (1.07), 228 (1.52), 248 (3.25) and 270 (3.66) nm; IR (KBr) ν_max_: 2982, 2867, 2248, 1720, 1716, 1384, 1142, 950 and 862 cm^−1^; ^1^H-NMR (500 MHz, CDCl_3_): *δ*_H_ 0.88 (3H, t, 7.0, H-17), 1.28 (8H, m, H-13, H-14, H-15, H-16), 1.36 (2H, m, H-12), 2.07 (3H, s, COCH_3_), 2.09 (3H, s, COCH_3_), 2.14 (2H, q, 7.0, H-11), 5.33 (1H, d, 10.0 Hz, H-1a), 5.46 (1H, ddt, 10.5, 8.5, 1.5Hz, H-9), 5.53 (1H, d, 17.5 Hz, H-1b), 5.66 (1H, ddt, 10.5, 7.0, 1.0 Hz, H-10), 5.84 (1H, ddd, 17.5, 10.0, 6.0 Hz, H-2), 5.90 (1H, dd, 6.0, 1.0 Hz, H-3), 6.13 (1H, d, 8.5 Hz, H-8). ^13^C-NMR (125 MHz, CDCl_3_): *δ*c 14.1 (C-17), 20.8 (COCH_3_), 20.9 (COCH_3_), 22.6 (C-15), 27.9 (C-11), 29.0 (C-12), 29.1 (C-14), 29.1 (C-13), 31.8 (C-16), 60.0 (C-8), 64.4 (C-3), 69.1 (C-6), 70.7 (C-5), 75.1 (C-4), 77.6 (C-7), 119.7 (C-1), 123.7 (C-10), 131.9 (C-9), 136.5 (C-2), 169.4 (C=O), 169.4 (C=O).

*(3S,8S,Z)-3-Hydroxyheptadeca-9-en-4,6-diyn-8-yl acetate* (**OHR-3**): yellowish oil (60 mg); 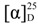 + 164.6° (*c* = 0.72, CHCl_3_); UV(CHCl_3_) λmax (log ξ): 203 (0.86), 224 (1.57), 242 (4.21), and 266 (4.58) nm; IR (KBr) ν_max_: 3385, 3025, 2946, 2825, 2230, 1715, 1580, 1420, 1232, 1060 and 896 cm^−1^; ^1^H-NMR (500 MHz, CDCl_3_): *δ*_H_ 0.88 (3H, t, 7.0, H-17), 1.00 (3H, t, 7.0, H-1),1.28 (8H, m, H-13, H-14, H-15, H-16), 1.38 (2H, m, H-12), 1.79 (2H, m, H-2), 2.07 (6H, s, COCH_3_) 2.13 (2H, dq, 7.0, 1.0 Hz, H-11), 4.37 (1H, t, 6.5 Hz, H-3), 5.47 (1H, ddt, 11.0, 8.5, 1.5 Hz, H-9), 5.65 (1H, ddt, 11.0, 7.5, 1.0 Hz, H-10), 6.13 (1H, d, 8.5 Hz, H-8). ^13^C-NMR (125 MHz, CDCl_3_): *δ*c 9.2 (C-1), 14.0 (C-17), 20.9 (COCH_3_), 22.6 (C-15), 27.8 (C-11), 27.8 (C-12), 29.1 (C-14), 29.2 (C-13), 30.6 (C-2), 31.7 (C-16), 60.1 (C-8), 64.0 (C-3), 68.9 (C-6), 69.4 (C-5), 75.9 (C-4), 80.9 (C-7), 123.9 (C-10), 136.3 (C-9), 169.5 (C=O).

*(3S,8S,Z)-8-Hydroxyheptadeca-9-en-4,6-diyn-3-yl acetate* (**OHR-4**): yellowish oil (55 mg); 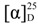 + 213.6° (*c* = 1.12, CHCl_3_); UV(CHCl_3_) λmax (log ξ): 205 (0.93), 222 (2.17), 245 (4.01), and 270 (4.27) nm; IR (KBr) ν_max_: 3360, 3010, 2936, 2860, 2252, 1722, 1596, 1440, 1210, 1060 and 912 cm^−1^; ^1^H-NMR (500 MHz, CDCl_3_): *δ*_H_ 0.88 (3H, t, 7.0, H-17), 1.02 (3H, t, 7.0, H-1), 1.28 (8H, m, H-13, H-14, H-15, H-16), 1.38 (2H, m, H-12), 1.79 (2H, m, H-2), 2.07 (3H, s, COCH_3_) 2.13 (2H, dq, 7.0, 1.0 Hz, H-11), 5.19 (1H, d, 8.0 Hz, H-8), 5.34 (1H, t, 6.5 Hz, H-3), 5.48 (1H, ddt, 11.0, 8.5, 1.5 Hz, H-9), 5.63 (1H, dt, 11.0, 7.5, 1.0 Hz, H-10). ^13^C-NMR (125 MHz, CDCl_3_): *δ*c 9.2 (C-1), 14.0 (C-17), 20.8 (COCH_3_), 22.6 (C-15), 27.7 (C-11), 27.9 (C-12), 29.1 (C-14), 29.2 (C-13), 29.3 (C-2), 31.8 (C-16), 58.7 (C-8), 65.3 (C-3), 69.2 (C-6), 69.3 (C-5), 76.0 (C-4), 79.4 (C-7), 127.8 (C-10), 134.7 (C-9), 169.5 (C=O).

*(3S,8S,Z)-3-Hydroxyheptadeca-1,9-dien-4,6-diyn-8-yl acetate* (**OHR-5**): yellowish oil (30 mg); 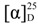 + 230.2° (*c* = 0.91, CHCl_3_); UV(CHCl_3_) λmax (log ξ): 210 (1.56), 228 (3.17), 242 (4.31), and 265 (4.45) nm; IR (KBr) ν_max_: 3422, 2981, 2258, 1716, 1584, 1432, 1226, 1030 and 990 cm^−1^; ^1^H-NMR (500 MHz, CDCl_3_): *δ*_H_ 0.89 (3H, t, 7.0, H-17), 1.27 (8H, m, H-13, H-14, H-15, H-16), 1.38 (2H, m, H-12), 2.07 (3H, s, COCH_3_), 2.14 (2H, q, 7.0, H-11), 4.93 (1H, d, 6.0 Hz, H-3), 5.27 (1H, d, 10.0 Hz, H-1a), 5.46 (1H, ddt, 10.5, 8.5, 1.5Hz, H-9), 5.50 (1H, d, 17.5 Hz, H-1b), 5.66 (1H, ddt, 10.5, 7.0, 1.0 Hz, H-10), 5.84 (1H, ddd, 17.5, 10.0, 6.0 Hz, H-2), 6.14 (1H, d, 8.5 Hz, H-8). ^13^C-NMR (125 MHz, CDCl_3_): *δ*c 14.1 (C-17), 20.9 (COCH_3_), 22.6 (C-15), 27.9 (C-11), 29.0 (C-12), 29.1 (C-14), 29.2 (C-13), 31.8 (C-16), 60.0 (C-8), 64.4 (C-3), 69.2 (C-6), 70.9 (C-5), 75.5 (C-4), 77.7 (C-7), 117.4 (C-1), 123.8 (C-10), 131.9 (C-9), 136.5 (C-2), 169.4 (C=O).

*(3S,8S,Z)-8-Hydroxyheptadeca-1,9-dien-4,6-diyn-3-yl acetate* (**OHR-6**): yellowish oil (45 mg); 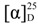 + 210.8° (*c* = 0.51, CHCl_3_); UV(CHCl_3_) λmax (log ξ): 208 (1.36), 226 (2.57), 248 (4.31), and 268 (4.48) nm; IR (KBr) ν_max_: 3380, 3012, 2252, 1718, 1560, 1482, 1216, 1024 and 990 cm^−1^; ^1^H-NMR (500 MHz, CDCl_3_): *δ*_H_ 0.89 (3H, t, 7.0, H-17), 1.27 (8H, m, H-13, H-14, H-15, H-16), 1.38 (2H, m, H-12), 2.09 (3H, s, COCH_3_), 2.11 (2H, q, 7.0, H-11), 5.19 (1H, d, 6.0 Hz, H-3), 5.33 (1H, d, 10.0 Hz, H-1a), 5.48 (1H, ddt, 10.5, 8.5, 1.5Hz, H-9), 5.51 (1H, d, 17.5 Hz, H-1b), 5.60 (1H, ddt, 10.5, 7.0, 1.0 Hz, H-10), 5.86 (1H, ddd, 17.5, 10.0, 6.0 Hz, H-2), 5.91 (1H, d, 8.5 Hz, H-8). ^13^C-NMR (125 MHz, CDCl_3_): *δ*c 14.0 (C-17), 20.8 (COCH_3_), 22.6 (C-15), 27.9 (C-11), 29.0 (C-12), 29.1 (C-14), 29.2 (C-13), 31.8 (C-16), 58.6 (C-8), 64.4 (C-3), 68.6 (C-6), 70.8 (C-5), 74.8 (C-4), 80.1 (C-7), 119.7 (C-1), 127.6 (C-10), 132.0 (C-9), 134.7 (C-2), 169.4 (C=O).

*(11S,16S,Z)-Octadeca-9-en-12,14-diyne-1,11,16-triyl triacetate* (**OHR-7**): yellowish oil (180 mg); 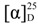 + 185.4° (*c* = 0.41, CHCl_3_); UV(CHCl_3_) λmax (log ξ): 205 (0.76), 223 (1.35), 240 (3.21), and 265 (4.05) nm; IR (KBr) ν_max_: 2985, 2258, 1723, 1716, 1585, 1477, 1238, 1028 and 954 cm^−1^; ^1^H-NMR (500 MHz, CDCl_3_): *δ*_H_ 1.00 (3 H, t, 7.5 Hz, H-18), 1.29 (8H, m, H-3, H-4, H-5, H-6), 1.37 (2H, m, H-7), 1.61 (2H, m, H-2), 1.79 (2H, m, H-17), 2.04 (3H, s, COCH_3_), 2.07 (6H, s, 2 × COCH_3_), 2.13 (2H, q, 7.5 Hz, H-8), 4.05 (2H, t, 7.0 Hz, H-1), 5.33 (1H, t, 6.5 Hz, H-16), 5.47 (1H, ddt, 10.6, 7.5, 1.5 Hz, H-9), 5.66 (1H, ddt, 10.6, 8.5, 1.5 Hz, H-10), 6.13 (1H, d, 8.5 Hz, H-11). ^13^C-NMR (125 MHz, CDCl_3_): *δ*c 9.2 (C-18), 20.8 (COCH_3_), 20.9 (COCH_3_), 21.0 (COCH_3_), 25.8 (C-2), 27.8 (C-8), 28.6 (C-6), 28.9 (C-5), 29.0 (C-4), 29.1 (C-3), 29.1 (C-7), 29.3 (C-17), 60.0 (C-11), 64.6 (C-16), 65.1 (C-1), 69.2 (C-13), 69.3 (C-14), 75.9 (C-15), 77.2 (C-12), 123.8 (C-10), 136.2 (C-9), 169.4 (C=O), 169.7 (C=O), 171.2 (C=O).

*(11S,16S,Z)-Octadeca-9,17-dien-12,14-diyne-1,11,16-triyl triacetate* (**OHR-8**): yellowish oil (175 mg); 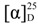 + 236.7° (*c* = 0.61, CHCl_3_); UV(CHCl_3_) λmax (log ξ): 210 (1.46), 228 (2.15), 246 (3.71), and 274 (4.65) nm; IR (KBr) ν_max_: 2968, 2257, 1720, 1718, 1562, 1457, 1318, 1016 and 946 cm^−1^; ^1^H-NMR (500 MHz, CDCl_3_): *δ*_H_ 1.29 (8H, m, H-3, H-4, H-5, H-6), 1.37 (2H, m, H-7), 1.62 (2H, m, H-2), 2.04 (3H, s, COCH3), 2.07 (3H, s, COCH3), 2.10 (3H, s, COCH3), 2.13 (2H, q, 7.5 Hz, H-8), 4.05 (2H t, 7.5 Hz, H-1), 5.33 (1H, d, 10.5 Hz, H-18a), 5.46 (1H, dt, 10.5, 7.5 Hz, Hz, H-9), 5.53 (1H, dd, 17.5, 1.5 Hz, H-18b), 5.66 (1H, ddt, 10.5, 7.5, 1.0Hz, H-10), 5.85 (1H, ddd, 17.0, 10.0, 1.5 Hz, H-17), 5.90 (1H, brd, 5.0 Hz, H-16), 6.13 (1H, d, 8.0 Hz, H-11). ^13^C-NMR (125 MHz, CDCl_3_): *δ*c 20.8 (COCH_3_), 20.9 (COCH_3_), 20.9 (COCH_3_), 25.8 (C-2), 27.8 (C-3), 28.6 (C-5), 29.0 (C-4), 29.0 (C-6), 29.1 (C-8), 29.2 (C-7), 60.0 (C-11), 64.3 (C-16), 64.6 (C-1), 69.1 (C-13), 70.7 (C-14), 75.1 (C-15), 76.7 (C-12), 119.7 (C-18), 123.7 (C-10), 131.9 (C-9), 136.3 (C-17), 169.3(C=O), 169.4 (C=O), 171.6 (C=O).

*(11S,16S,Z)-16-Hydroxyoctadeca-9,17-dien-12,14-diyne-1,16-diyl diacetate* (**OHR-9**): yellowish oil (110 mg); 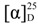 + 285.2° (*c* = 1.01, CHCl_3_); UV(CHCl_3_) λmax (log ξ): 208 (1.40), 226 (3.15), 245 (4.81), and 268 (4.55) nm; IR (KBr) ν_max_: 3348, 3014, 2988, 2254, 1722, 1710, 1486, 1380, 1258, 1016 and 885 cm^−1^; ^1^H-NMR (500 MHz, CDCl_3_): *δ*_H_ 1.29 (8H, m, H-3, H-4, H-5, H-6), 1.37 (2H, m, H-7), 1.62 (2H, m, H-2), 2.04 (3H, s, COCH3), 2.07 (3H, s, COCH3), 2.13 (2H, q, 7.5 Hz, H-8), 4.05 (2H t, 7.5 Hz, H-1), 5.19 (1H, d, 8.0 Hz, H-11), 5.34 (1H, d, 10.5 Hz, H-18a), 5.50 (1H, dt, 10.5, 7.5 Hz, Hz, H-9), 5.52 (1H, dd, 17.5, 1.5 Hz, H-18b), 5.62 (1H, ddt, 10.5, 7.5, 1.0Hz, H-10), 5.86 (1H, ddd, 17.0, 10.0, 1.5 Hz, H-17), 5.90 (1H, brd, 5.0 Hz, H-16). ^13^C-NMR (125 MHz, CDCl_3_): *δ*c 20.8 (COCH_3_), 20.9 (COCH_3_), 25.8 (C-2), 27.8 (C-3), 28.6 (C-5), 29.0 (C-4), 29.0 (C-6), 29.1 (C-8), 29.2 (C-7), 58.6 (C-11), 64.3 (C-16), 64.6 (C-1), 68.6 (C-13), 70.8 (C-14), 74.9 (C-15), 80.1 (C-12), 119.7 (C-18), 127.7 (C-10), 132.0 (C-9), 134.6 (C-17), 169.4(C=O), 171.3 (C=O).

*(11S,16S,Z)-16-Hydroxyoctadeca-9,17-dien-12,14-diyne-1,11-diyl diacetate* (**OHR-10**): yellowish oil (110 mg); 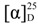 + 285.2° (*c* = 1.01, CHCl_3_); UV(CHCl_3_) λmax (log ξ): 208 (1.40), 226 (3.15), 245 (4.81), and 268 (4.55) nm; IR (KBr) ν_max_: 3348, 3014, 2988, 2254, 1722, 1710, 1486, 1380, 1258, 1016 and 885 cm^−1^; ^1^H-NMR (500 MHz, CDCl_3_): *δ*_H_ 1.26 (8H, m, H-3, H-4, H-5, H-6), 1.41 (2H, m, H-7), 1.61 (2H, m, H-2), 2.04 (3H, s, COCH3), 2.07 (3H, s, COCH3), 2.13 (2H, q, 7.5 Hz, H-8), 4.05 (2H t, 7.5 Hz, H-1), 4.94 (1H, brd, 5.0 Hz, H-16), 5.25 (1H, d, 10.5 Hz, H-18a), 5.50 (1H, dt, 10.5, 7.5 Hz, Hz, H-9), 5.55 (1H, dd, 17.5, 1.5 Hz, H-18b), 5.65 (1H, ddt, 10.5, 7.5, 1.0Hz, H-10), 5.96 (1H, ddd, 17.0, 10.0, 1.5 Hz, H-17), 6.13 (1H, d, 8.0 Hz, H-11). ^13^C-NMR (125 MHz, CDCl_3_): *δ*c 20.8 (COCH_3_), 21.0 (COCH_3_), 25.8 (C-2), 27.8 (C-3), 28.6 (C-5), 29.0 (C-4), 29.0 (C-6), 29.1 (C-8), 30.4 (C-7), 58.6 (C-11), 64.3 (C-16), 64.6 (C-1), 68.6 (C-13), 70.8 (C-14), 74.9 (C-15), 80.1 (C-12), 117.3 (C-18), 123.9 (C-10), 130.8 (C-9), 136.2 (C-17), 169.5 (C=O), 171.3 (C=O).

*(11S,16S,Z)-11-Hydroxyoctadeca-9-en-12,14-diyne-1,16-diyl diacetate* (**OHR-11**): yellowish oil (30 mg); 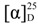 + 225.4° (*c* = 1.01, CHCl_3_); UV(CHCl_3_) λmax (log ξ): 205 (0.76), 223 (1.35), 240 (3.21), and 265 (4.05) nm; IR (KBr) ν_max_: 3310, 2985, 2258, 1723, 1716, 1585, 1477, 1238, 1028 and 954 cm^−1^; ^1^H-NMR (500 MHz, CDCl_3_): *δ*_H_ 0.89 (3 H, t, 7.5 Hz, H-18), 1.28 (8H, m, H-3, H-4, H-5, H-6), 1.38 (2H, m, H-7), 1.62 (2H, m, H-2), 1.78 (2H, m, H-17), 2.04 (3H, s, COCH_3_), 2.09 (3, s, COCH_3_), 2.13 (2H, q, 7.5 Hz, H-8), 4.05 (2H, t, 7.0 Hz, H-1), 5.33 (1H, t, 6.5 Hz, H-16), 5.47 (1H, ddt, 10.6, 7.5, 1.5 Hz, H-9), 5.66 (1H, ddt, 10.6, 8.5, 1.5 Hz, H-10), 5.90 (1H, d, 8.5 Hz, H-11). ^13^C-NMR (125 MHz, CDCl_3_): *δ*c 9.2 (C-18), 20.8 (COCH_3_), 20.9 (COCH_3_), 25.8 (C-2), 27.8 (C-8), 28.6 (C-6), 28.9 (C-5), 29.0 (C-4), 29.1 (C-3), 29.1 (C-7), 29.3 (C-17), 60.0 (C-11), 64.6 (C-16), 65.1 (C-1), 69.2 (C-13), 69.5 (C-14), 75.6 (C-15), 77.6 (C-12), 126.1 (C-10), 134.3 (C-9), 169.4 (C=O), 171.2 (C=O). 

*(11S,16S,Z)-16-Hydroxyoctadeca-9-en-12,14-diyne-1,11-diyl diacetate* (**OHR-12**): yellowish oil (80 mg); 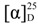 + 241.4° (*c* = 1.21, CHCl_3_); UV(CHCl_3_) λmax (log ξ): 204 (0.86), 225 (1.25), 242 (3.61), and 266 (4.15) nm; IR (KBr) ν_max_: 3326, 2986, 2252, 1720, 1715, 1565, 1447, 1232, 1120 and 880 cm^−1^; ^1^H-NMR (500 MHz, CDCl_3_): *δ*_H_ 1.00 (3 H, t, 7.5 Hz, H-18), 1.29 (8H, m, H-3, H-4, H-5, H-6), 1.38 (2H, m, H-7), 1.62 (2H, m, H-2), 1.74 (2H, m, H-17), 2.04 (3H, s, COCH_3_), 2.07 (3, s, COCH_3_), 2.15 (2H, q, 7.5 Hz, H-8), 4.06 (2H, t, 7.0 Hz, H-1), 4.37 (1H, t, 6.5 Hz, H-16), 5.48 (1H, ddt, 10.6, 7.5, 1.5 Hz, H-9), 5.66 (1H, ddt, 10.6, 8.5, 1.5 Hz, H-10), 6.13 (1H, d, 8.5 Hz, H-11). ^13^C-NMR (125 MHz, CDCl_3_): *δ*c 9.2 (C-18), 20.8 (COCH_3_), 20.9 (COCH_3_), 25.8 (C-2), 27.6 (C-8), 28.6 (C-6), 28.9 (C-5), 29.0 (C-4), 29.1 (C-3), 29.3 (C-7), 30.6(C-17), 58.6 (C-11), 64.0 (C-16), 65.4 (C-1), 68.7 (C-13), 69.5 (C-14), 75.7 (C-15), 81.1 (C-12), 124.0 (C-10), 136.1 (C-9), 169.4 (C=O), 171.4 (C=O).

*(3S,8S,Z)-18-Hydroxyoctadeca-9-en-4,6-diyne-3,8-diyl diacetate* (**OHR-13**): yellowish oil (20 mg); 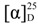 + 168.6° (*c* = 0.50, CHCl_3_); UV(CHCl_3_) λmax (log ξ): 206 (0.56), 226 (1.55), 242 (2.86), and 265 (3.84) nm; IR (KBr) ν_max_: 3410, 3020, 2252, 1718, 1714, 1562, 1460, 1235, 1022 and 950 cm^−1^; ^1^H-NMR (500 MHz, CDCl_3_): *δ*_H_ 1.00 (3 H, t, 7.5 Hz, H-1), 1.28 (8H, m, H-13, H-14, H-15, H-16), 1.37 (2H, m, H-12), 1.61 (2H, m, H-17), 1.79 (2H, m, H-2), 2.04 (3H, s, COCH_3_), 2.09 (3H, s, COCH_3_), 2.13 (2H, q, 7.5 Hz, H-11), 4.08 (2H, t, 7.0 Hz, H-18), 5.33 (1H, t, 6.5 Hz, H-3), 5.47 (1H, ddt, 10.6, 7.5, 1.5 Hz, H-10), 5.66 (1H, ddt, 10.6, 8.5, 1.5 Hz, H-9), 6.13 (1H, d, 8.5 Hz, H-8). ^13^C-NMR (125 MHz, CDCl_3_): *δ*c 9.2 (C-1), 20.8 (COCH_3_), 21.0 (COCH_3_), 25.8 (C-17), 27.8 (C-11), 28.6 (C-13), 28.9 (C-14), 29.0 (C-15), 29.1 (C-16), 29.1 (C-12), 29.3 (C-2), 60.0 (C-8), 64.6 (C-3), 65.1 (C-18), 69.1 (C-6), 69.2 (C-5), 75.7 (C-4), 77.5 (C-7), 123.6 (C-9), 136.1 (C-10), 169.4 (C=O), 169.7 (C=O).

*(3S,8S,Z)-18-Hydroxyoctadeca-1,9-dien-4,6-diyne-3,8-diyl diacetate* (**OHR-14**): yellowish oil (30 mg); 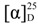 + 236.7° (*c* = 0.61, CHCl_3_); UV(CHCl_3_) λmax (log ξ): 210 (1.46), 228 (2.15), 246 (3.71), and 274 (4.65) nm; IR (KBr) ν_max_: 3380, 3010, 2252, 1718, 1713, 1568, 1438, 1310, 1016 and 890 cm^−1^; ^1^H-NMR (500 MHz, CDCl_3_): *δ*_H_ 1.31 (8H, m, H-13, H-14, H-15, H-16), 1.38 (2H, m, H-12), 1.61 (2H, m, H-17), 2.04 (3H, s, COCH3), 2.07 (3H, s, COCH3), 2.14 (2H, q, 7.5 Hz, H-11), 4.09 (2H t, 7.5 Hz, H-17), 5.33 (1H, d, 10.5 Hz, H-1a), 5.46 (1H, dt, 10.5, 7.5 Hz, Hz, H-10), 5.53 (1H, dd, 17.5, 1.5 Hz, H-1b), 5.66 (1H, ddt, 10.5, 7.5, 1.0Hz, H-9), 5.85 (1H, ddd, 17.0, 10.0, 1.5 Hz, H-2), 5.90 (1H, brd, 5.0 Hz, H-3), 6.13 (1H, d, 8.0 Hz, H-8). ^13^C-NMR (125 MHz, CDCl_3_): *δ*c 20.8 (COCH_3_), 20.9 (COCH_3_), 25.8 (C-17), 27.8 (C-16), 28.6 (C-14), 29.0 (C-15), 29.0 (C-13), 29.1 (C-11), 29.2 (C-12), 60.0 (C-8), 64.3 (C-3), 64.6 (C-18), 69.1 (C-6), 70.7 (C-5), 75.1 (C-4), 76.7 (C-7), 119.7 (C-1), 123.7 (C-9), 131.9 (C-10), 136.3 (C-2), 169.3(C=O), 169.4 (C=O).

*(3S,8S,Z)-3,18-Dihydroxyoctadeca-1,9-dien-4,6-diyn-8-yl acetate* (**OHR-15**): yellowish oil (16 mg); 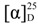 + 216.8° (*c* = 0.26, CHCl_3_); UV(CHCl_3_) λmax (log ξ): 206 (0.86), 228 (2.35), 247 (3.75), and 270 (4.25) nm; IR (KBr) ν_max_: 3460, 3010, 2250, 1716, 1562, 1442, 1320, 1038 and 860 cm^−1^; ^1^H-NMR (500 MHz, CDCl_3_): *δ*_H_ 1.31 (8H, m, H-13, H-14, H-15, H-16), 1.37 (2H, m, H-12), 1.61 (2H, m, H-17), 2.07 (3H, s, COCH3), 2.14 (2H, q, 7.5 Hz, H-11), 4.09 (2H t, 7.5 Hz, H-17), 4.33 (1H, brd, 5.0 Hz, H-3), 5.33 (1H, d, 10.5 Hz, H-1a), 5.48 (1H, dt, 10.5, 7.5 Hz, Hz, H-10), 5.52 (1H, dd, 17.5, 1.5 Hz, H-1b), 5.66 (1H, ddt, 10.5, 7.5, 1.0Hz, H-9), 5.92 (1H, ddd, 17.0, 10.0, 1.5 Hz, H-2), 6.13 (1H, d, 8.0 Hz, H-8). ^13^C-NMR (125 MHz, CDCl_3_): 20.9 (COCH_3_), 25.8 (C-17), 27.8 (C-16), 28.6 (C-14), 29.0 (C-15), 29.0 (C-13), 29.1 (C-11), 29.2 (C-12), 61.3 (C-8), 63.3 (C-3), 64.6 (C-18), 69.1 (C-6), 70.6 (C-5), 75.6 (C-4), 79.8 (C-7), 119.7 (C-1), 127.8 (C-10), 132.1 (C-9), 136.4 (C-2), 169.4 (C=O).

*(3S,8S,Z)-3,18-Dihydroxyoctadeca-1,9-dien-4,6-diyn-3-yl acetate* (**OHR-16**): yellowish oil (20 mg); 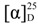 + 176.4° (*c* = 0.16, CHCl_3_); UV(CHCl_3_) λmax (log ξ): 203 (0.75), 225 (2.10), 248 (2.65), and 266 (4.86) nm; IR (KBr) ν_max_ :3410, 3022, 2258, 1720, 1582, 1432, 1360, 1024 and 860 cm^−1^; ^1^H-NMR (500 MHz, CDCl_3_): *δ*_H_ 1.31 (8H, m, H-13, H-14, H-15, H-16), 1.37 (2H, m, H-12), 1.61 (2H, m, H-17), 2.04 (3H, s, COCH3), 2.14 (2H, q, 7.5 Hz, H-11), 4.09 (2H t, 7.5 Hz, H-17), 5.13 (1H, d, 8.0 Hz, H-8) 5.33 (1H, d, 10.5 Hz, H-1a), 5.48 (1H, dt, 10.5, 7.5 Hz, Hz, H-10), 5.52 (1H, dd, 17.5, 1.5 Hz, H-1b), 5.68 (1H, ddt, 10.5, 7.5, 1.0Hz, H-9), 5.90 (1H, brd, 5.0 Hz, H-3), 5.92 (1H, ddd, 17.0, 10.0, 1.5 Hz, H-2). ^13^C-NMR (125 MHz, CDCl_3_): 20.9 (COCH_3_), 25.8 (C-17), 27.8 (C-16), 28.6 (C-14), 29.0 (C-15), 29.0 (C-13), 29.1 (C-11), 29.2 (C-12), 61.3 (C-8), 63.3 (C-3), 64.6 (C-18), 69.1 (C-6), 70.6 (C-5), 75.5 (C-4), 77.8 (C-7), 117.2 (C-1), 129.2 (C-10), 134.4 (C-9), 136.4 (C-2), 169.3 (C=O).

### 3.7. Anticancer Assay

#### 3.7.1. Cancer Cell Lines and Culture

The human breast cancer cell lines MCF-7, MDA-MB-231, human hepatocellular liver cell line HepG2 and human lung carcinoma epithelial cells A549 were purchased from American Type Culture Collection (ATCC, Manassas, VA, USA). Normal human hepatic LO2 cells were obtained from the Institute of Cell Biology, Academic Sinica (Shanghai, China). The cells were grown in an atmosphere of 5% CO_2 _at 37 °C in RPMI 1640 medium supplemented with 10% fetal bovine serum, 100 U/mL penicillin and 100 mg/mL streptomycin.

#### 3.7.2. Measurement of Cell Viability

The viability of cells was measured by a colorimetric MTT assay. Briefly, 1 × 10^4 ^cells/well in 96-well microplates were exposed to different concentration of polyynes and their acetylated derivatives respectively for 24 h incubation, following addition of 20 μL MTT solution (4 mg/mL) to each well for another 4 h incubation at 37 °C. Finally, the medium was removed and replaced with 100 μL dimethyl sulfoxide which was added to dissolve the dye crystal presented in cells. The absorbance was recorded at 570 nm using a microplate reader (1420 Multilabel counter victor^3^, Perkin-Elmer, Waltham, MA, USA). The results were expressed as ratio of absorbance between treatments and control cells (solvent vehicle set at 100%). Four replicate wells were tested per assay which was repeated three times.

#### 3.7.3. Hoechst Staining

MDA-MB-231 cells were seeded in the flat-bottomed 6-well plates and treated with **OHR-1**, **-2**, **-3** and **-5**. Cells treated with solvent vehicle served as controls. In 12 h after treatment, cells were fixed in 4% polyoxymethylene solution for 30 min and then washed with PBS. The fixed cells were incubated with Hoechst 33342 for 15 min at room temperature in the dark and then washed with PBS twice, which were finally observed under a fluorescence microscope (Convert Fluorescence Microsope Axiovert 200, HAL 100, HBO 100, Carl Zeiss MicroImaging Co., Ltd., Oberkochen, Germany) at a magnification of ×400.

#### 3.7.4. Measurement of Mitochondrial Membrane Potential

To assess the mitochondrial membrane potential, Δψm, JC-1 staining was used to exhibit potential dependent accumulation in mitochondria, indicating reversibly change color from green to red fluorescence as the membrane potential increases. On the other hand, in apoptotic or unhealthy cells with low Δψm, JC-1 showed an increasing ratio of green to red fluorescence intensity. The MDA-MB-231 cells were treated with **OHR-1**, **-2**, **-3** and **-5** for 24 h in a humidified atmosphere (37 °C with 5% CO_2_). The cells were collected and responded in 0.5 mL culture medium with JC-1 dye (1 μg/mL) for 20 min at 37 °C in dark. Fluorescence was monitored by both flow cytometry (BD Biosciences, San Jose, CA, USA) and fluorescence microscope. The Δψm were indicated by the rato of green to red fluorescence intensity. The data were expressed as ratio of Δψm between treatments and control cells (solvent vehicle set at 100%).

#### 3.7.5. Cell Cycle Analysis by Flow Cytometry

Cells were seeded in 6-well tissue culture plates and treated with DMSO control or different concentration of **OHR-1**, **-2**, **-3** and **-5** for 24 h. Then, the cells were harvested and fixed gently with cold 70% ethanol at −20 °C overnight. Finally, the cells were washed with PBS and re-suspended in 300 μL of propidium iodide (PI) stain solution (20 μg/mL PI and 8 μg/mL DNase-free RNase) for 20 min at room temperature in the dark. The cells were analyzed with a flow cytometry and Mod Fit LT 3.0 software (Variety Software House, Topsham, ME, USA). For each measurement, at least 20,000 cells were counted.

#### 3.7.6. Statistical Analysis

Data were presented as mean ± SEM of at least three independent experiments performed in quadruplicates for each sample. A one-way analysis of variance (ANOVA) followed by Turkey *post-hoc* test (GraphPad Prism 6.0, San Diego, CA, USA) determined whether the results had statistical significance between groups. Values of *p* < 0.05 were considered as statistical significance.

## 4. Conclusions

Six polyynes, namely falcarindiol (**OH-1**), oplopandiol (**OH-2**), (11*S*,16*S*,9*Z*)-9,17-octadecadiene-12,14-diyne-1,11,16-triol,1-acetate (**OH-3**), oplopandiol acetate (**OH-4**), oplopantriol A (**OH-5**) and oplopantriol B (**OH-6**), had been isolated and identified from the root bark of *Oplopanax horridus* (Devil’s Club) ‒ a natural dietary supplement and medicinal plant in North America. Sixteen acetylated polyynes were synthesized from the isolated polyynes. The polyynes presented different inhibitory effects on four human cancer cell lines. Primary structure–activity analysis showed that the observed inhibitions were influenced mostly by the terminal ethenyl and hydroxyl groups and acylations of the polyynes. This study showed that acetylated polyynes retained the same anti-proliferative activity mechanism as non-acetylated polyynes. Moreover, the safety evaluation on the usage of *O. horridus* is worthy of study in future.
