# Correction: Huang, W.-H., *et al.* Anticancer Activities of Polyynes from the Root Bark of *Oplopanax horridus* and Their Acetylated Derivatives. *Molecules* 2014, *19*, 6142-6162

**DOI:** 10.3390/molecules20045438

**Published:** 2015-03-26

**Authors:** Lan-Zhen Meng, Wei-Hua Huang, Chong-Zhi Wang, Chun-Su Yuan, Shao-Ping Li

**Affiliations:** 1 State Key Laboratory of Quality Research in Chinese Medicine, Institute of Chinese Medical Sciences, University of Macau, Avenida da Universidade, Macau, China; E-Mails: Meng-lz@hotmail.com (L.-Z.M.); endeavor34852@aliyun.com (W.-H.H.); 2 Tang Center for Herbal Medicine Research, The Pritzker School of Medicine, University of Chicago, 5841 South Maryland Avenue, MC 4028, Chicago, IL 60637, USA; E-Mail: CWang@dacc.uchicago.edu

We wish to make the following changes to the published article [1], agreed upon by all authors: Lan-Zhen Meng and Shao-Ping Li have been added as co-authors, Li Shao and Hong-Hao Zhou have been removed as co-authors, and the acknowledgements have been altered to more appropriately recognize support and funding. Author contributions are corrected accordingly. We apologize to readers of *Molecules* for any inconvenience caused by these changes.

The corrected author list, acknowledgements, author contributions are provided below:

Lan-Zhen Meng ^1,†^, Wei-Hua Huang ^1,†^, Chong-Zhi Wang ^2^, Chun-Su Yuan ^2,^* and Shao-Ping Li ^1,^*

^1^ State Key Laboratory of Quality Research in Chinese Medicine, Institute of Chinese Medical Sciences, University of Macau, Avenida da Universidade, Macau, China; E-Mails: Meng-lz@hotmail.com (L.-Z.M.); endeavor34852@aliyun.com (W.-H.H.)
^2^ Tang Center for Herbal Medicine Research, The Pritzker School of Medicine, University of Chicago, 5841 South Maryland Avenue, MC 4028, Chicago, IL 60637, USA; E-Mail: CWang@dacc.uchicago.edu
^†^ These authors contributed equally to this work.
* Authors to whom correspondence should be addressed; E-Mails: SPLi@umac.mo (S.-P.L.); CYuan@dacc.uchicago.edu (C.-S.Y.); Tel.: +853-8822-4692 (S.-P.L.); +1-773-702-1916 (C.-S.Y.); Fax: +853-2884-1358 (S.-P.L.); +1-773-834-0601 (C.-S.Y.).
